# Maternal Sedentary Behavior and Physical Activity across Pregnancy and Early Childhood Motor Development

**DOI:** 10.3390/children8070549

**Published:** 2021-06-25

**Authors:** Melissa A. Jones, Kara M. Whitaker, Sharon E. Taverno Ross, Kelliann Davis, Klaus Libertus, Bethany Barone Gibbs

**Affiliations:** 1 Department of Health and Human Physiology, University of Iowa, Iowa City, IA 52242, USA; kara-whitaker@uiowa.edu; 2 Department of Epidemiology, University of Iowa, Iowa City, IA 52241, USA; 3 Department of Health and Human Development, University of Pittsburgh, Pittsburgh, PA 15260, USA; seross@pitt.edu (S.E.T.R.); kelli.davis@pitt.edu (K.D.); bbarone@pitt.edu (B.B.G.); 4 Department of Psychology, University of Pittsburgh, Pittsburgh, PA 15260, USA; Klaus.libertus@pitt.edu

**Keywords:** DOHaD, accelerometry, maternal–child health, epidemiology

## Abstract

Early childhood motor development is an important indicator of short- and long-term health. In utero exposures impact offspring health across the lifespan; however, whether maternal activity during pregnancy may impact early childhood motor development remains unknown. This prospective cohort study measured the motor development skills of *n* = 70 children born to mothers from a previously conducted cohort study which objectively measured activity profile, (sedentary behavior (SED) and moderate-to-vigorous intensity physical activity (MVPA), across pregnancy. Mothers reported the motor development of their child using the Early Motor Questionnaire (EMQ). Linear regression models examined associations between maternal activity profile and EMQ scores. Maternal SED and MVPA were analyzed in two ways: trimester-specific and across pregnancy using trajectory groups. Children were 12–30 months of age, majority white (82%), and 52% male. Maternal SED during pregnancy was not associated with any EMQ domains (gross motor, fine motor, and perception action). Higher maternal MVPA, across pregnancy by trajectory group and in the first and second trimesters, was significantly associated with moderate-sized effects of more advanced fine motor and perception action scores. Higher MVPA in early pregnancy appears to be related to more advanced early childhood motor development. Therefore, maternal MVPA may be a modifiable behavior by which short- and long-term offspring health may be impacted.

## 1. Introduction

The Developmental Origins of Health and Disease (DOHaD) theory focuses on the fetal environment as the earliest determinant of short- and long-term health risks in offspring [1]. Poorer health in childhood generally tracks into poorer health in adulthood [2,3], making it of critical importance to prevent the early development of adult disease. Therefore, an improved understanding of modifiable behaviors during pregnancy that may impact the fetal environment and offspring health is needed.

One potential modifiable behavior is maternal activity profile, including sedentary behavior (SED), defined as low-intensity behaviors in a sitting, reclining, or lying position [4], and moderate-to-vigorous intensity physical activity (MVPA) during pregnancy. These behaviors affect the fetal environment through nutrient delivery, placental alterations, and energy metabolism [5,6]. There is a growing body of evidence to suggest negative impacts of high SED during pregnancy on maternal [7] and child health [8]. Additionally, the benefits of MVPA during pregnancy for the mother and baby, including reduced risk for gestational diabetes, preeclampsia, excessive gestational weight gain and improved fetal growth, are well documented [9,10]. However, less is known about whether the effects of these behaviors on the fetus during pregnancy persist into early childhood. Therefore, more research is needed to understand how maternal SED and MVPA may impact early childhood health.

One important indicator of health in early childhood is motor development. Motor development is foundational for children’s physical, social, and psychological health [11]. Poorer or delayed motor development is related to an increased risk for obesity, lower levels of MVPA, and lower cardiorespiratory fitness across childhood [12,13,14]. While the importance of motor development skills in early childhood is well established, how the intrauterine environment may impact the development of motor skills in childhood remains unknown. Birth size, often used a proxy measure of sufficiency of the intrauterine environment, may be associated with motor development. In one previous study, children born with a lower birth weight had lower motor development scores between 2 and 47 months of age than those born with normal birth weight [15]. Within the context of the DOHaD theory, this suggests that in utero exposures may impact childhood motor skills and subsequent fitness levels, obesity, and cardiometabolic health risk. However, the association between maternal activity during pregnancy and early childhood motor development is unclear.

Though the activity profile during pregnancy is plausibly associated with early childhood motor development, research investigating this relationship is extremely limited by the use of self-reported maternal MVPA, failure to examine maternal SED, or study designs that only capture late pregnancy activity. Further, available studies have produced mixed findings [16,17,18,19]. This is an important research gap as maternal activity during pregnancy is a modifiable behavior that could potentially improve short- and long-term offspring health. Therefore, the primary purpose of this study was to examine the associations between objectively measured SED and MVPA across all three trimesters of pregnancy with early childhood motor development skills. A secondary objective was to examine whether size at birth explained these associations. We hypothesized that lower maternal SED and higher maternal MVPA would be associated with more advanced motor development skills in the offspring and that adjustment for birth size would attenuate these associations.

## 2. Materials and Methods

The present study added follow-up assessments on the children of women enrolled in the MOnitoring Movement and Health (MoM Health) study, which was a prospective cohort study. The MoM Health study included three study visits, one in each trimester of pregnancy, where women received two movement monitors (described below) to objectively measure SED and MVPA. The purpose of this follow-up study was to examine health outcomes in the children born to mothers in the parent study in early childhood (between the ages of 12 and 30 months). To be eligible for the parent study, women had to be between the ages of 18 and 45, <14 weeks pregnant, not taking any blood pressure or glucose lowering medications, and able to walk a half of a mile and climb two flights of stairs. Participants with ≥1 trimester of valid objective activity monitoring and a singleton live birth (*n* = 103) were contacted for follow-up recruitment and were deemed eligible if they had custody of their child and the child did not have any congenital or chromosol abnormalities that would affect growth or development.

In the follow-up study, which is the focus of this manuscript, mothers were asked to complete an electronic questionnaire battery. Informed consent was obtained from all participants involved in this study and all procedures were approved by the University of Pittsburgh Institutional Review Board.

### 2.1. Maternal–Child Characteristics

A standard demographics questionnaire was used to collect the child’s age, race, and primary feeding type (i.e., breastfeeding, formula fed) along with maternal education and household income. Questionnaires were completed electronically using REDCap (Research Electronic Data Capture) web-based software. Birth length and weight measures were abstracted from medical records and converted into body mass index (BMI) z-scores using the STATA World Health Organization z-score calculator plug-in [20].

### 2.2. Objective Measurement of SED and MVPA

Maternal activity profile data were initially collected in the parent study and included measurement in each trimester of pregnancy using gold-standard, objective measurement methodology described in detail elsewhere [7]. The activPAL3 micro accelerometer (PALtechnologies, Glasgow, Scotland) was used to measure SED with a 24 h wear protocol. Participants were instructed to only remove this thigh-mounted monitor for swimming and to complete a diary to document all sleep and non-wear periods. Data from the activPAL were exported in event format and trained research staff removed all sleep and non-wear times [21,22]. Actigraph GT3X accelerometers (Actigraph, Pensacola, FL, USA) worn on an elastic belt on the right hip and below the gravid abdomen were used to measure MVPA. Participants were instructed to wear this monitor during all waking hours, only to be removed for sleep or water activities (bathing or swimming). Data were analyzed using Actilife v6 12.2 software using Freedson 2011 VM cutpoints for classifying MVPA [23]. Participants were instructed to wear monitors concurrently for 7 days, and data were considered valid if worn for ≥10 h on ≥4 days [22,24].

**Figure 1:**
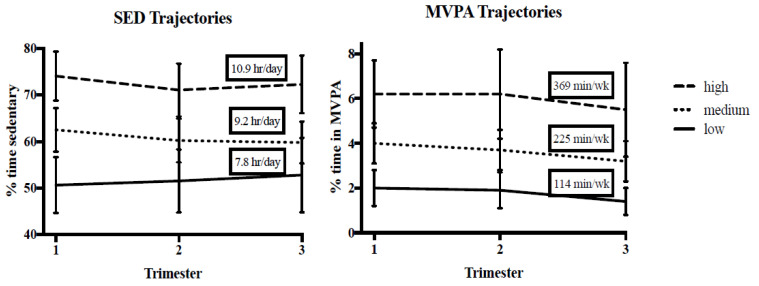
Maternal SED and MVPA trajectory groups across pregnancy trimesters.

Group-based trajectory analysis was used to describe activity patterns across pregnancy [25]. Trajectory groups were auto-generated separately for SED and MVPA and best fit was selected based on the Bayesian criterion index (BIC), greatest percentage of participants placed in groups with posterior probability of ≥70%, and clinical relevance of derived groups. High, medium, and low groups were generated separately for SED and MVPA and women were assigned to the group that most closely fit their activity patterns. Mean percent time spent in SED and MVPA across pregnancy in the high, medium, and low trajectory groups are displayed in Figure 1 and further details of these groups have been previously reported elsewhere [7].

### 2.3. Motor Development

The Early Motor Questionnaire (EMQ) [26] was used to measure motor development by parent report. Participants responded to questions about their child’s behaviors at the time of questionnaire completion using a 5-point scale ranging from −2 (sure child does not show behavior) to +2 (sure child shows behavior). The questions provide a composite score in three domains: gross motor (possible range: −98 to +98), fine motor (possible range: −96 to +96), and perception-action (possible range: −62 to +62). The three domain scores correspond to full body movements and large muscle group control (gross motor), small muscle groups and ability to grasp, hold, or manipulate objects (fine motor), and a child’s ability to use their senses to gather information and respond to the world around them (perception action) [27]. An instructional video accompanied the questionnaire to aid in the proper completion of the EMQ. The EMQ is widely used to measure parent-reported motor development in early childhood and has high concurrent validity with gold-standard, examiner-administered motor development measurement (gross motor: *r* = 0.97, fine motor: *r* = 0.91, perception action: *r* = 0.91) [26]. Due to wide variations in age within our sample, including *n* = 26 children >24 months of age, models used raw EMQ scores as the dependent variable with adjustment for age using a linear spline with an inflection point at 24 months. This methodological choice reflects the fact that the EMQ score is expected to increase more steeply up to age 24 months and then be more stable after 24 months.

### 2.4. Statistical Analysis

Participant characteristics were summarized overall and by SED and MVPA trajectory groups using mean (SD) for continuous variables and frequencies for categorical variables. Differences in participant characteristics by trajectory groups were examined using either Kruskal-Wallis for continuous variables or Fisher’s exact test for categorical variables. Maternal SED and MVPA were examined using two approaches, trimester-specific and across pregnancy by trajectory group. Linear regression models, adjusted for age splines, examined the relationship between trimester-specific and trajectory groups maternal SED and MVPA, with the three EMQ score domains (gross motor, fine motor, and perception action). From the age-adjusted linear regression models examining SED and MVPA trajectories, predicted least square mean EMQ scores were calculated and used to illustrate predicted averages with age adjustment by maternal activity trajectories. Semipartial correlations were used to assess the effect size (meaningfulness) of associations, in which effects < 0.2 are considered weak, 0.2–0.5 are moderate, and >0.5 are strong [28]. To examine the secondary objective, all linear regression models were repeated, including adjustment for BMI z-score at birth. Differences in significance and magnitude of associations were qualitatively assessed before and after adjustment for BMI z-score at birth to explore consistency of associations.

Of the *n* = 103 participants contacted for this follow-up study, *n* = 74 replied and consented to participate. Four participants did not complete the EMQ, resulting in an analytical sample of *n* = 70. Women that responded and enrolled in the present study were significantly younger, more highly educated, and less racially diverse than the parent study sample. Due to our small sample size and resulting limited ability to adjust for covariates, participant characteristics (e.g., income, pre-pregnancy BMI, race, age, education, child feeding type) that could potentially confound our analyses were evaluated in all statistical models. These sensitivity analyses were conducted whereby the potentially confounding characteristics were tested for influence in all statistical models one at a time.

With our *n* = 70, age splines explained roughly 45% of the variance in linear regression models. Post-hoc power calculations determined that we had 80% power to detect additional covariates that explained 7% or more of the remaining variance with an alpha level of 0.05.

## 3. Results

Children (*n* = 70) were between the ages of 13 and 30 months (mean: 21.6 ± 5.2 months) at the time of follow-up, and 53% male, primarily white (82%), and mostly had mothers who were highly educated (57% had a masters or doctoral degree). SED and MVPA trajectory group membership was independent (χ^2^ *p* = 0.703). Demographic and clinical characteristics are summarized in Table 1 overall and by maternal SED and MVPA trajectory. No characteristics significantly differed across either activity trajectory group.

### 3.1. Early Motor Questionnaire and Maternal SED and MVPA Trajectories

**Figure 2:**
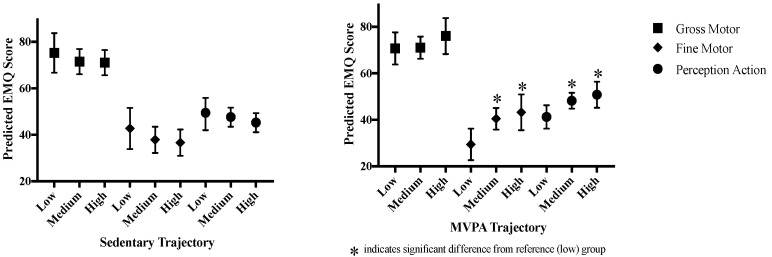
Predicted EMQ scores by maternal SED and MVPA trajectory groups.

Predicted least square mean scores for each EMQ domain by maternal SED and MVPA trajectories were estimated using age-adjusted linear regression models and are presented in Figure 2. Linear regression models examining associations between maternal SED and MVPA trajectories with EMQ domains found that predicted fine motor and perception-action scores were higher in the medium or high maternal MVPA trajectory groups compared to low maternal MVPA and did not differ by SED trajectory groups. MVPA trajectories explained 7.2% and 7.1% of the variance in fine motor and perception-action scores, respectively. Compared to the children with mothers in the low MVPA group, fine motor scores were 11.00 and 13.76 points higher in the children with mothers in the medium or high groups, respectively (medium group 95% CI: 2.67, 19.33, high group 95% CI: 3.51, 24.00). Children with mothers in the medium or high MVPA groups had perception-action scores that were 7.02 and 9.56 points higher as compared with children of mothers in the low group, respectively (medium group 95% CI: 0.92, 13.12, high group 95% CI: 2.07, 17.06). All significant differences in scores correspond to a moderate effect sizes with semipartial correlations ranging from 0.24 to 0.28. Results from linear regression models examining associations between EMQ with maternal SED and MVPA trajectories are presented in Table S1.

### 3.2. Early Motor Questionnaire and Trimester-Specific Maternal SED and MVPA

Results from linear regression models examining associations of trimester-specific SED and MVPA with EMQ domain scores are presented in Table 2. Consistent with the trajectory models, maternal SED in any trimester was not associated with early childhood gross motor, fine motor, or perception-action scores. Higher maternal MVPA in the first and second trimesters was significantly associated with higher fine motor scores (first trimester std ß: 4.33, 95% CI: 0.81, 7.84, second trimester std ß: 3.72, 95% CI: 0.10, 7.33). Higher MVPA was significantly related to higher perception-action scores in the first (std ß: 3.78, 95% CI: 1.29, 6.27) and second (std ß: 2.87, 95% CI: 0.27, 5.47) trimesters. MVPA explained 5.1% and 7.0% of the variance in fine motor and perception-action scores, respectively.

### 3.3. Early Motor Questionnaire and Maternal Activity with and without Additional Adjustments

Participants that did not have both valid EMQ data and BMI z-score data were excluded from analyses with additional adjustment for BMI z-score at birth, resulting in an analytical sample of *n* = 59 for these analyses. Associations between maternal activity trajectories and EMQ scores, before and after adjustment for infant birth BMI z-score, are presented in Table 3. High maternal MVPA trajectory was significantly associated with higher fine motor score with and without adjustment for BMI z-score at birth. Higher perception-action score was significantly associated with high maternal MVPA trajectory prior to adjustment for BMI z-score at birth; though the magnitude of association was slightly strengthened, the association was no longer statistically significant after adjustment.

Associations between trimester-specific maternal activity and EMQ scores, before and after adjustment for infant birth BMI z-score, are presented in Table 4. With this smaller sample, higher maternal MVPA was associated with higher fine motor and perception action scores in the first trimester only. The addition of BMI z-score at birth to models did not impact the significance or magnitude of associations.

Relevant participant characteristics and concurrent adjustment for SED and MVPA were added to each trajectory and trimester-specific model to test for influence. Inclusion of maternal age, pre-pregnancy BMI, race, child feeding type, maternal education, household income, and SED (in MVPA models) or MVPA (in SED models) one at a time did not change the statistical significance or magnitude of association in statistical models. Results from these sensitivity analyses are displayed in the supplemental materials, trajectory models (Table S2) and trimester-specific models (Table S3).

## 4. Discussion

This study was conducted to better understand how the maternal activity profile across pregnancy may relate to early childhood motor development. The main findings are that maternal MVPA, but not SED, during pregnancy is related to early childhood motor development. Higher levels of MVPA, across pregnancy and in the first and second trimester, were associated with more advanced fine motor (small muscle group control) and perception-action (physical response to visual stimuli) scores between 13 and 30 months of age. Motor development skills in early childhood are critical for social and psychological functioning and health [11]. Poorer motor development may have long-term consequences as it has been related to an increased risk for obesity and lower fitness levels later in life [12,13]. These findings indicate that engaging in MVPA during pregnancy may have short- and long-term effects on offspring health.

This study contributes to an important research gap by examining maternal SED during pregnancy and how it relates to early childhood motor development. No previous research has examined this association; therefore, our findings in which maternal SED, across pregnancy or in any trimester, did not relate to early childhood gross motor, fine motor, or perception-action cannot be put into a broader context for comparison. However, our data do offer novel evidence that maternal SED does not appear to impact early childhood motor development due to the trivial magnitude and non-significance of associations observed.

Few studies have examined the association between maternal MVPA in pregnancy and child motor development. Of the existing studies, measurement of developmental skills varies greatly, including cognitive, language, motor, and intelligence domains, making it difficult to compare findings [17,18,19,29]. In one observational cohort study, most similar to our study, and including 528 pregnant women, maternal leisure time physical activity was self-reported in each trimester and development was measured using the Bayley Scales of Infant and Toddler Development in children at 1- and 2-year follow-up. This study found no difference in the motor development score across maternal physical activity levels in children at either follow-up timepoint. This cohort study differs from ours by measurement methodology for both physical activity and child development. Our study objectively measured MVPA and included all domains of MVPA accumulated throughout the day, while the comparison study used self-reported leisure time physical activity only. Including all activity accumulated across the day may have provided a more sensitive and biologically relevant measure of overall physical activity habits, which in turn may be more strongly related to motor development than leisure time physical activity alone. Another difference in methodology was the other study’s measure of motor development, where this outcome was directly assessed by trained research staff using a stronger measure of motor development than our parent-reported questionnaire.

The association between maternal MVPA and child motor development has also been tested experimentally, with most studies finding no significant association. One structured exercise program included one in-person 60 min exercise session and two at-home 45 min sessions of aerobic and strength exercise per week between 20 and 36 weeks of pregnancy. This study then compared the developmental scores of children (intervention: *n* = 164, control: *n* = 115) at 7 years of age using the ‘Five-to-Fifteen’ motor development questionnaire. No differences were found in fine or gross motor score domains in children born to mothers in the intervention compared to the control groups [18]. Another randomized controlled trial which also included one in-person and two at-home 45 min sessions per week but limited to aerobic exercise only (intervention: *n* = 188, control *n* = 148), measured development using the Bayley Scales of Infant and Toddler Development in children at 18 months old and also found no significant differences in overall motor development scores between intervention and control groups. However, while non-significant, children of mothers in the exercise intervention had lower motor scores than controls [17]. Lastly, and contrary to other findings, a small, supervised exercise trial including three 50 min moderate-intensity aerobic exercise sessions per week with 27 intervention and 33 control participants measured the motor development of children using the Peabody Developmental Motor Scales at one-month follow-up. This study found that, at one month old, children born to intervention mothers had significantly higher locomotion scores than controls [19]. Scores were also higher for stationary and gross motor domains compared to controls but were not statistically significant. These studies differ from ours because of the experimental manipulation of MVPA levels beginning in the second trimester. Our observational design, beginning in the first trimester, may capture more habitual exercise and effects of early pregnancy activity, which may explain the different findings. Further, the varying age of follow-up and motor development measurement tools in the existing literature makes comparison difficult. Taken together, habitual exercise may have a positive impact on early childhood motor development, while introducing activity during pregnancy may have no effect or only short-term benefits [19] on motor development. Further investigation is needed to better understand the short- and long-term developmental implications of MVPA during pregnancy.

While we cannot say for certain that the mechanisms by which MVPA in pregnancy may lead to more advanced motor development in early childhood as we did not formally study these, we propose two possibilities. The first is related to the Developmental Origins of Health and Disease theory. This theory would posit that higher levels of MVPA, primarily in early pregnancy, could aid in improved fetal development. Early pregnancy is when the structure and function of organ systems are being developed, as opposed to late pregnancy, when fetal growth is primarily body fat development. Maternal MVPA during pregnancy is associated with improved nutrient sensing and vascularization of the placenta [5,6]. Improved nutrient transport in early pregnancy may relate to more optimal development of musculoskeletal and organ systems in utero, which may then allow for more advanced motor development in childhood. This could be consistent with our findings in which adjustment for BMI z-score at birth did not explain the associations between maternal MVPA and fine motor and perception-action scores. This suggests that any effect of MVPA in late pregnancy would impact fat deposition and soft tissue growth, which was no longer associated with motor development in our analysis. Therefore, the early pregnancy effects on musculoskeletal and organ system development related to MVPA may be a plausible mechanism by which higher MVPA relates to improved motor development.

The second proposed mechanism is postnatal exposure. Our study was observational in nature and, therefore, likely captured habitual exercise. Although not measured in this study, those with high levels of MVPA during pregnancy may be more likely to be physically active postpartum [30]. Further, we propose that women who are more active themselves may also be more active with their child, which would encourage motor developmental behaviors. However, evidence supporting a direct relationship between parental activity and child motor development is limited. More advanced motor development has been associated with higher levels of physical activity in children [14] and physically active parents are more likely to have physically active children [31]. One study found that higher levels of paternal, but not maternal, accelerometer-measured MVPA were associated with improved motor development in 846 preschool-aged children [32]. In contrast, another observational study actually found that maternal self-reported physical activity was related to poorer object control, a domain of motor development in the Test for Gross Motor Development-2 [33]. Though plausible, current data do not draw a clear link between maternal physical activity in pregnancy or postpartum with motor development in children. Further studies are needed to elucidate the mechanism by which maternal MVPA may relate to motor development. Future research should examine environmental and social determinants of childhood motor development as well as the physiological differences or biomarkers related to improved motor development to differentiate between the effects of nature versus nurture.

### Strengths and Limitations

The use of objective, gold-standard measurement of maternal SED and MVPA is a major strength of this study. The prospective design, with multiple measurement timepoints, allowed for analysis by trimester and patterns across pregnancy and established temporality between pre- and post-natal outcomes. Further, the observational design of the study may capture the effects of early pregnancy activity and habitual activity patterns rather than only specific domains of SED or MVPA (i.e., leisure time, occupational) or the introduction of new behaviors by experimental manipulation, often in the second trimester or later.

While this study has several strengths, findings should be interpreted with caution due to several limitations, including the small sample and possibility for type-I error inflation due to multiple comparisons. The small sample size and limited racial and sociodemographic variability limited our ability to adjust for potential confounders and, while adjusting for these one at a time did not alter our findings, we may have lacked power to detect differences. An additional limitation was the use of parent-reported motor development using the Early Motor Questionnaire. While this tool is considered valid and reliable compared to objectively measured motor development tools, there is the risk of bias or misrepresentation of motor development ability with parent report. Over- or under-reporting of motor development by the mothers could have influenced our results. Further, this questionnaire was also collected with a variable follow-up interval (from 13 to 30 months), and sometimes outside of the optimized window for the motor development assessment tool that we used (after 24 months old). Further, due to the observational nature of this study, we cannot infer causality from our findings. As we did not experimentally manipulate maternal activity patterns during pregnancy, we cannot say for certain that changing SED or MVPA would elicit the same effects. Lastly, our sample participated in low levels of vigorous intensity activity, which limited our ability to examine the associations of higher activity intensities with offspring motor development. However, despite these limitations, our findings do offer preliminary support for and inspire future study of the association between maternal activity profiles and early childhood motor development.

## 5. Conclusions

Higher levels of MVPA, specifically in early pregnancy, were associated with more advanced early childhood motor development. This finding adds to a robust body of evidence on the benefits of MVPA during pregnancy. While maternal SED does not appear to relate to motor development, more evidence is needed as our study has limitations in sample size and rigor of motor development assessment and was the first to examine this association. As motor development skills in early childhood are critical for a child’s short- and long-term physical, social, and psychological health, our findings support maternal MVPA as a modifiable behavior by which health across the lifespan may be impacted.

## Figures and Tables

**Table 1:** Participant characteristics overall and by maternal SED and MVPA trajectory groups.

		Sedentary Trajectory (*n*)				MVPA Trajectory (*n*)			
	Overall (70)	Low (12)	Med (29)	High (29)	*p*	Low (18)	Med (38)	High (14)	*p*
**Median** **(IQR 25%, 75%)**									
Child age, months	22.7 (16.7, 26.5)	25.4 (21.9, 28.0)	20.7 (16.8, 26.5)	22.8 (16.6, 26.0)	0.327	23.6 (15.1, 26.56)	22.7 (16.8, 26.2)	22.8 (16.8, 26.09)	0.964
Gestational age at birth, weeks	38.3 (38.4, 40.0)	39.6 (39.14, 39.71)	39.0 (38.1, 40.0)	39.4 (38.3, 40.0)	0.679	39.1 (38.6, 39.6)	39.7 (38.3, 40.0)	38.9 (37.0, 39.7)	0.285
Maternal pre-pregnancy BMI, kg/m^2^	21.5 (21.5, 29.5)	27.5 (23.3, 21.9)	24.3 (19.9, 27.4)	23.9 (21.9, 25.7)	0.199	25.2 (21.0, 31.6)	24.0 (21.7, 30.8)	24.0 (21.7, 27.5)	0.845
Maternal EPDS score *	2.0 (1.0, 5.0)	3.5 (0.0, 8.0)	1.0 (0.0, 4.0)	3.0 (2.0, 5.0)	0.153	4.0 (2.0, 5.0)	2.0 (0.0, 5.0)	2.0 (0.5, 5.5)	0.469
***n* (%)**									
**Sex**					0.059				0.470
Male	37 (53)	4 (33)	13 (45)	20 (69)		12 (66)	18 (47)	7 (50)	
Female	33 (47)	8 (67)	16 (55)	9 (31)		6 (33)	20 (53)	7 (50)	
**Feeding type**					0.926				0.757
Exclusively breastfed	36 (51)	7 (58)	15 (52)	14 (48)		9 (50)	20 (53)	7 (50)	
Partial breastfeeding	31 (44)	5 (42)	12 (41)	14 (48)		9 (50)	15 (39)	7 (50)	
Exclusively formula-fed	3 (4)	0	2 (7)	1 (4)		0	3 (8)	0	
**Maternal Education**					0.214				0.157
High school or less	1 (1)	0	0	1 (2)		1 (5)	0	0	
Some college or training	11 (15)	4 (33)	4 (14)	3 (10)		5 (28)	3 (8)	3 (21)	
College graduate	18 (27)	4 (33)	5 (17)	9 (31)		5 (28)	11 (29)	2 (14)	
Masters/Doctoral	40 (57)	4 (33)	20 (69)	16 (55)		7 (39)	25 (63)	9 (64)	
**Household Income**					0.188				0.448
<50,000	6 (8)	3 (25)	2 (7)	1 (3)		1 (6)	4 (11)	1 (7)	
50,000–75,000	8 (11)	2 (17)	4 (14)	2 (7)		3 (17)	4 (11)	1 (7)	
>75,000	53 (76)	6 (50)	22 (76)	25 (86)		12 (67)	30 (78)	11 (80)	
Don’t know/refused to answer	3 (4)	1 (8)	1 (3)	1 (4)		2 (11)	0	1 (7)	
**Race**					0.871				0.538
White	58 (82)	9 (75)	25 (86)	24 (83)		13 (72)	32 (84)	13 (93)	
Black	6 (9)	2 (17)	2 (7)	2 (7)		2 (11)	3 (8)	1 (7)	
Asian	6 (9)	1 (8)	2 (7)	3 (10)		3 (17)	3 (8)	0	

EPDS: Edinburgh Postpartum Depression Score, BMI: body mass index. * Fewer observations available for EPDS score (*n* = 49).

**Table 2:** Results from linear regression models examining association of trimester-specific SED and MVPA with EMQ domains.

	Trimester 1		Trimester 2		Trimester 3	
	Std. ß	95% CI	Std. ß	95% CI	Std. ß	95% CI
SED						
Gross Motor	−2.10	5.69, 1.49	−2.50	−6.08, 1.07	−1.55	−4.85, 1.96
Fine Motor	0.26	−3.55, 4.06	−1.92	−5.66, 1.82	−1.96	−5.42, 1.79
Perception-Action	−1.35	−4.08, 1.38	−1.36	−4.09, 1.36	−0.79	−3.36, 1.89
MVPA						
Gross Motor	1.90	−1.58, 5.38	0.29	−3.27, 3.84	−1.03	−4.74, 2.68
Fine Motor	4.33	0.81, 7.84	3.72	0.10, 7.33	2.70	−1.20, 6.60
Perception-Action	3.78	1.29, 6.27	2.87	0.27, 5.47	1.42	−1.47, 4.32

Std. ß: Coefficient representing change in EMQ score per 1 SD change SED or MVPA. SD SED: Trimester 1: 87.2 Trimester 2: 75.8 Trimester 3: 81.2 min. SD MVPA: Trimester 1: 16.6 Trimester 2: 17.3 Trimester 3: 17.3 min.

**Table 3:** Associations between activity trajectories and EMQ Scores with and without adjustment for BMI z-score at birth.

	Low		Medium		High	
	Coef.	95% CI	Coef.	95% CI	Coef.	95% CI
SED Trajectories						
Gross Motor						
Model 1	Reference	-	−2.07	−14.21, 10.07	−1.63	−13.75, 10.49
Model 2	Reference	-	−1.96	−14.33, 10.51	−1.43	−14.42, 10.51
Fine Motor						
Model 1	Reference	-	−4.65	−15.64, 6.33	−3.39	−14.35, 7.57
Model 2	Reference	-	−5.19	−16.44, 6.06	−4.37	−16.02, 7.27
Perception-Action						
Model 1	Reference	-	−1.01	−10.12, 8.09	−2.77	−11.85, 6.32
Model 2	Reference	-	−1.59	−10.89, 7.71	−3.83	−13.45, 5.80
MVPA Trajectories						
Gross Motor						
Model 1	Reference	-	−0.95	−10.65, 8.74	3.57	−8.40, 15.54
Model 2	Reference	-	−0.70	−10.61, 9.20	4.09	−8.42, 16.61
Fine Motor						
Model 1	Reference	-	7.22	−1.26, 15.70	11.60	1.13, 22.07
Model 2	Reference	-	7.36	−1.31, 16.03	11.91	0.96, 22.86
Perception-Action						
Model 1	Reference	-	5.38	−1.68, 12.45	8.80	0.08, 17.52
Model 2	Reference	-	5.43	−1.79, 12.65	8.90	−0.22, 18.03

Model 1 includes adjustment for age splines. Model 2 includes adjustment for age splines and BMI z-score at birth.

**Table 4:** Associations between trimester-specific activity and EMQ scores with and without adjustment for BMI z-score at birth.

	Trimester 1		Trimester 2		Trimester 3	
	Std. Coef.	95% CI	Std. Coef.	95% CI	Std. Coef.	95% CI
SED						
Gross Motor						
Model 1	−1.46	−5.67, 2.79	−2.00	−6.33, 2.33	−0.52	−4.68, 3.63
Model 2	−1.59	−6.0, 2.85	−1.99	−6.38, 2.41	−0.80	−5.09, 3.48
Fine Motor						
Model 1	0.47	−3.40, 4.34	0.03	−3.91, 3.97	−0.12	−3.95, 3.73
Model 2	0.31	−3.76, 4.38	−0.07	−4.07, 3.92	−0.29	−4.25, 3.67
Perception-Action						
Model 1	−1.36	−4.53, 1.81	−0.68	−3.95, 2.59	−0.01	−3.18. 3.17
Model 2	−1.70	−5.02, 1.62	−0.79	−4.10, 2.52	−0.28	−3.54, 2.98
MVPA						
Gross Motor						
Model 1	1.20	−2.92, 5.32	−1.21	−5.57, 3.15	−2.27	−5.46, 2.68
Model 2	1.32	−2.93, 5.54	−1.30	−6.24, 3.64	−1.39	−6.62, 2.09
Fine Motor						
Model 1	3.72	0.09, 7.34	3.24	−0.64, 7.12	2.04	−1.71, 5.79
Model 2	3.72	0.00, 7.44	3.62	−0.76, 8.01	1.98	−2.09, 6.04
Perception-Action						
Model 1	3.77	0.83, 6.71	2.91	−0.27, 6.10	1.07	−2.11, 4.24
Model 2	3.78	0.76, 6.79	3.19	−0.41, 6.79	0.79	−2.63, 4.23

Model 1 includes adjustment for age splines. Model 2 includes adjustment for age splines and BMI z-score at birth. Std. Coef: Coefficient representing change in EMQ score per 1 SD change SED or MVPA. SD SED: Trimester 1: 87.2 Trimester 2: 75.8 Trimester 3: 81.2 min. SD MVPA: Trimester 1: 16.6 Trimester 2: 17.3 Trimester 3: 17.3 min.

## Data Availability

Supporting data is not publicly available due to patient confidentiality.

## Supplementary Materials

The following are available online at https://www.mdpi.com/article/10.3390/children8070549/s1, Table S1: Association of age-adjusted and age-standardized EMQ domains with maternal SED and MVPA by trajectory groups., Table S2: Sensitivity analyses examining associations of SED and MVPA trajectories with and without adjustment for potential confounders., Table S3: Sensitivity analyses examining associations of trimester-specific SED and MVPA with and without adjustment for potential confounders.

## References

[1] Barker D.J. (2007). The origins of the developmental origins theory. J. Intern. Med..

[2] Singh A.S., Mulder C., Twisk J.W., Van Mechelen W., Chinapaw M.J. (2008). Tracking of childhood overweight into adulthood: A systematic review of the literature. Obes. Rev..

[3] Kelly R.K., Thomson R., Smith K.J., Dwyer T., Venn A., Magnussen C.G. (2015). Factors affecting tracking of blood pressure from childhood to adulthood: The childhood determinants of adult health study. J. Pediatrics.

[4] Bames J., Behrens T.K., Benden M.E., Biddle S., Bond D., Brassard P., Brown H., Carr L., Carson V., Chaput J. (2012). Letter to the Editor: Standardized use of the terms “sedentary” and “sedentary behaviours”. Appl. Physiol. Nutr. Metab. Physiol. Appl. Nutr. Metab..

[5] Barakat R., Cordero Y., Coteron J., Luaces M., Montejo R. (2012). Exercise during pregnancy improves maternal glucose screen at 24–28 weeks: A randomised controlled trial. Br. J. Sports Med..

[6] Brett K., Ferraro Z., Holcik M., Adamo K. (2015). Prenatal physical activity and diet composition affect the expression of nutrient transporters and mTOR signaling molecules in the human placenta. Placenta.

[7] Gibbs B.B., Jones M.A., Jakicic J.M., Jeyabalan A., Whitaker K.M., Catov J.M. (2021). Objectively Measured Sedentary Behavior and Physical Activity Across 3 Trimesters of Pregnancy: The Monitoring Movement and Health Study. J. Phys. Act. Health.

[8] Jones M.A., Catov J.M., Jeyabalan A., Whitaker K.M., Barone Gibbs B. (2021). Sedentary behaviour and physical activity across pregnancy and birth outcomes. Paediatr. Perinat. Epidemiol..

[9] Birsner M.L., Gyamfi-Bannerman C. (2020). Physical Activity and Exercise During Pregnancy and the Postpartum Period. ACOG Committee Opinion No. 804. Obstet. Gynecol..

[10] Pastorino S., Bishop T., Crozier S., Granström C., Kordas K., Küpers L., O’Brien E., Polanska K., Sauder K., Zafarmand M. (2019). Associations between maternal physical activity in early and late pregnancy and offspring birth size: Remote federated individual level meta-analysis from eight cohort studies. BJOG Int. J. Obstet. Gynaecol..

[11] Gallahue D., Ozmun J. (2006). Understanding Motor Development: Infants, Children, Adolescents, Adults.

[12] Barnett L.M., Van Beurden E., Morgan P.J., Brooks L.O., Beard J.R. (2009). Childhood motor skill proficiency as a predictor of adolescent physical activity. J. Adolesc. Health.

[13] Graf C., Koch B., Kretschmann-Kandel E., Falkowski G., Christ H., Coburger S., Lehmacher W., Bjarnason-Wehrens B., Platen P., Tokarski W. (2004). Correlation between BMI, leisure habits and motor abilities in childhood (CHILT-project). Int. J. Obes..

[14] Lubans D.R., Morgan P.J., Cliff D.P., Barnett L.M., Okely A.D. (2010). Fundamental Movement Skills in Children and Adolescents. Sports Med..

[15] Hediger M.L., Overpeck M.D., Ruan W.J., Troendle J.F. (2002). Birthweight and gestational age effects on motor and social development. Paediatr. Perinat. Epidemiol..

[16] Álvarez-Bueno C., Cavero-Redondo I., Sánchez-López M., Garrido-Miguel M., Martínez-Hortelano J.A., Martínez-Vizcaíno V. (2018). Pregnancy leisure physical activity and children’s neurodevelopment: A narrative review. BJOG Int. J. Obstet. Gynaecol..

[17] Hellenes O.M., Vik T., Løhaugen G.C., Salvesen K.Å., Stafne S.N., Mørkved S., Evensen K.A.I. (2015). Regular moderate exercise during pregnancy does not have an adverse effect on the neurodevelopment of the child. Acta Paediatr..

[18] Ellingsen M.S., Pettersen A., Stafne S.N., Mørkved S., Salvesen K.Å., Evensen K.A.I. (2020). Neurodevelopmental outcome in 7-year-old children is not affected by exercise during pregnancy: Follow up of a multicentre randomised controlled trial. BJOG: BJOG Int. J. Obstet. Gynaecol..

[19] McMillan A.G., May L.E., Gaines G.G., Isler C., Kuehn D. (2019). Effects of Aerobic Exercise during Pregnancy on 1-Month Infant Neuromotor Skills. Med. Sci. Sports Exerc..

[20] Leroy J. (2011). ZSCORE06: Stata Module to Calculate Anthropometric Z-Scores Using the 2006 WHO Child Growth Standards.

[21] Barone Gibbs B., Kline C.E. (2018). When does sedentary behavior become sleep? A proposed framework for classifying activity during sleep-wake transitions. Int. J. Behav. Nutr. Phys. Act..

[22] Edwardson C.L., Winkler E.A., Bodicoat D.H., Yates T., Davies M., Dunstan D.W., Healy G.N. (2017). Considerations when using the activPAL monitor in field-based research with adult populations. J. Sport Health Sci..

[23] Sasaki J.E., John D., Freedson P.S. (2011). Validation and comparison of ActiGraph activity monitors. J. Sci. Med. Sport.

[24] Matthews C.E., Hagstromer M., Pober D.M., Bowles H.R. (2012). Best practices for using physical activity monitors in population-based research. Med. Sci. Sports Exerc..

[25] Jones B.L., Nagin D.S. (2013). A note on a Stata plugin for estimating group-based trajectory models. Sociol. Methods Res..

[26] Libertus K., Landa R.J. (2013). The Early Motor Questionnaire (EMQ): A parental report measure of early motor development. Infant Behav. Dev..

[27] Department of Health and Human Services Effective Practice Guide: Perceptual, Motor, and Physical Development. Early Childhood Learning and Knowledge Center. https://eclkc.ohs.acf.hhs.gov/school-readiness/effective-practice-guides/perceptual-motor-physical-development.

[28] Acock A.C. (2008). A Gentle Introduction to Stata.

[29] Polańska K., Muszyński P., Sobala W., Dziewirska E., Merecz-Kot D., Hanke W. (2015). Maternal lifestyle during pregnancy and child psychomotor development—Polish Mother and Child Cohort study. Early Hum. Dev..

[30] Borodulin K., Evenson K.R., Herring A.H. (2009). Physical activity patterns during pregnancy through postpartum. BMC Women’s Health.

[31] Hinkley T., Crawford D., Salmon J., Okely A.D., Hesketh K. (2008). Preschool children and physical activity: A review of correlates. Am. J. Prev. Med..

[32] Cools W., De Martelaer K., Samaey C., Andries C. (2011). Fundamental movement skill performance of preschool children in relation to family context. J. Sports Sci..

[33] Barnett L.M., Hnatiuk J.A., Salmon J., Hesketh K.D. (2019). Modifiable factors which predict children’s gross motor competence: A prospective cohort study. Int. J. Behav. Nutr. Phys. Act..

