# Supplementary material for: Maternal Sedentary Behavior and Physical Activity across Pregnancy and Early Childhood Motor Development
Source: Children (Basel). 2021 Jun 25;8(7):549. doi: 10.3390/children8070549 (PMC8304240; doi:10.3390/children8070549)
Supplement: Supplementary file 1 [file children-08-00549-s001.zip › children-1248427-supplementary.pdf]

Supplementary Materials

Table S1. Association of age-adjusted and age-standardized EMQ domains with maternal SED and MVPA by trajectory groups.

	**Low**		**Medium**			**High**		
	**Coef.**	**95% CI**	**Coef.**	**95% CI**	**ßstdXY**	**Coef.**	**95% CI**	**ßstdXY**
SED Trajectories								
Gross Motor	Reference	-	−3.74	−13.86, 6.40	0.094	−4.11	−14.27, 6.06	0.104
Fine Motor	Reference	-	−4.87	−15.43, 5.69	0.123	−6.11	−16.70, 4.48	0.155
Perception–Action	Reference	-	−1.82	−9.46, 5.83	0.063	−4.18	−11.85, 3.49	0.146
MVPA Trajectories								
Gross Motor	Reference	-	0.31	−8.10, 8.73	0.008	5.33	−5.01, 15.67	0.102
Fine Motor	Reference	-	11.00	2.66, 19.32	0.282	13.76	3.51, 24.00	0.283
Perception–Action	Reference	-	7.02	0.92, 13.12	0.247	9.56	2.07, 17.06	0.271

SED: sedentary behavior; MVPA: moderate-to-vigorous intensity physical activity

ßstdXY: Semipartial correlation represents effect size in which ß < 0.20 is weak, 0.20 < ß<0.50 is moderate, and ß > 0.50 is strong. Models include adjustment for age splines with a 24-month inflection point.

Table S2. Sensitivity analyses examining associations of SED and MVPA trajectories with and without adjustment for potential confounders.

	**Low**		**Medium**		**High**	
	**Coef.**	**95% CI**	**Coef.**	**95% CI**	**Coef.**	**95% CI**
**SED Trajectories**						
**Gross Motor**						
Primary Model	Reference	-	−3.72	−13.87, 6.40	−4.11	−14.27,6.06
Maternal Age	Reference	-	−3.12	−13.26, 7.02	−2.49	−12.94,7.96
Race	Reference	-	−2.89	−13.17, 7.39	−3.50	−13.80, 6.82
Household Income	Reference	-	−2.45	−13.09, 8.19	−2.29	−13.15, 8.58
Feeding type	Reference	-	−3.91	−14.38, 6.41	−4.07	−14.38, 6.25
Maternal Pre-Pregnancy BMI	Reference	-	−1.63	−11.77, 8.52	−2.48	−12.57, 7.60
Education	Reference	-	−1.19	−11.60, 9.21	−2.14	−12.56, 8.27
MVPA Trajectories	Reference	-	−3.37	−13.60, 6.86	−3.94	−14.27, 6.39
**Fine Motor**						
Primary Model	Reference	-	−4.87	−15.43, 5.69	−6.11	−16.70, 4.48
Maternal Age	Reference	-	−5.78	−16.22, 4.66	−8.48	−19.24, 2.27
Race	Reference	-	−5.06	−15.89, 5.77	−6.25	−17.11, 4.61
Household Income	Reference	-	−4.51	−15.46, 6.45	−5.63	−16.82, 5.57
Feeding Type	Reference	-	−4.92	−15.70, 5.86	−6.07	−16.84, 4.71
Maternal Pre-Pregnancy BMI	Reference	-	−5.33	−16.20, 5.54	−6.46	−17.27, 4.34
Education	Reference	-	−4.42	−15.44, 6.60	−6.60	−17.64, 4.43
MVPA Trajectories	Reference	-	−3.71	−13.84, 6.41	−4.15	−14.38, 6.07
**Perception–Action**						
Primary Model	Reference	-	−1.82	−9.46, 5.83	−4.18	−11.85, 3.49
Maternal Age	Reference	-	−1.69	−9.43, 6.05	−3.86	−11.83, 4.12
Race	Reference	-	−3.29	−10.69, 4.11	−5.50	−12.92, 1.92
Household Income	Reference	-	−3.56	−11.18, 4.06	−6.59	−14.38, 1.20

Feeding Type	Reference	-	−1.33	−9.03, 6.37	−3.87	−11.57, 3.82
Maternal Pre-Pregnancy BMI	Reference	-	−3.14	−10.87, 4.58	−5.20	−12.88, 2.48
Education	Reference	-	−3.53	−11.45, 4.40	−5.64	−13.58, 2.29
MVPA Trajectories	Reference	-	−1.02	−8.43, 6.39	−2.93	−10.41, 4.55
**MVPA Trajectories**						
**Gross Motor**						
Primary Model	Reference	-	0.32	−8.10, 8.73	5.33	−5.01, 15.67
Maternal Age	Reference	-	0.28	−8.09, 8.65	5.00	−5.29, 15.30
Race	Reference	-	0.92	−7.54, 9.38	6.52	−4.03,17.07
Household Income	Reference	-	0.26	−8.50, 9.01	5.79	−4.70, 16.27
Feeding Type	Reference	-	−0.20	−8.84, 8.44	5.31	−5.15, 15.78
Maternal Pre-Pregnancy BMI	Reference	-	1.32	−6.90, 9.56	6.41	−3.70, 16.51
Education	Reference	-	3.07	−5.62, 11.76	7.38	−3.05, 17.81
SED Trajectory	Reference	-	−0.09	−8.69, 8.52	4.91	−5.61, 15.42
**Fine Motor**						
Primary Model	Reference	-	11.00	2.55, 19.32	13.76	3.51, 24.00
Maternal Age	Reference	-	11.03	2.80, 19.27	14.15	4.02, 24.28
Race	Reference	-	11.18	2.66, 19.69	14.17	3.55, 24.80
Household Income	Reference	-	9.74	1.07, 18.41	13.48	3.09, 23.87
Feeding Type	Reference	-	11.04	2.45, 19.64	13.75	3.34, 24.16
Maternal Pre-Pregnancy BMI	Reference	-	11.07	2.62, 19.52	13.84	3.46, 24.21
Education	Reference	-	12.38	3.67, 21.09	15.83	5.38, 26.27
SED Trajectory	Reference	-	10.58	2.06, 19.10	13.31	2.90, 23.72
**Perception–Action**						
Primary Model	Reference	-	7.02	0.92, 13.12	9.56	2.07, 17.06
Maternal Age	Reference	-	7.01	0.88, 13.14	9.46	1.91, 17.01
Race	Reference	-	6.67	0.73, 12.60	9.11	1.70, 16.51
Household Income	Reference	-	5.59	0.63, 11.81	8.63	1.18, 16.08
Feeding Type	Reference	-	7.50	1.32, 13.65	9.53	2.06, 17.01
Maternal Pre-Pregnancy BMI	Reference	-	6.60	0.48, 12.72	9.12	1.6, 16.63
Education	Reference	-	6.42	−0.09, 12.94	9.23	1.42, 17.04
SED Trajectory	Reference	-	6.61	0.38, 12.84	9.29	1.68, 16.90

Each potential confounding variable was added to the primary model one at a time (adjusted for age splines only). SED: sedentary behavior; MVPA: moderate-to-vigorous intensity physical activity; BMI: body mass index (kg/m^2).

Table S3. Sensitivity analyses examining associations of trimester-specific SED and MVPA with and without adjustment for potential confounders.

	**Trimester 1**		**Trimester 2**		**Trimester 3**	
	**Std. ß**	**95% CI**	**Std. ß.**	**95% CI**	**Std. ß**	**95% CI**
**SED**						
**Gross Motor**						
Primary Model	−2.10	−5.69, 1.49	−2.50	−6.08, 1.07	−1.44	−4.85,1.96
Maternal Age	−1.51	−5.25, 2.23	−2.06	−5.70, 1.58	−0.91	−4.38, 2.56
Race	−2.14	−5.74, 1.47	−2.31	−5.92, 1.29	−1.33	−4.78, 2.12
Household Income	−1.34	−5.26, 2.57	−1.88	−5.71, 1.95	−0.88	−4.44, 2.68
Feeding Type	−1.96	−5.63, 1.72	−2.41	−6.06, 1.25	−1.47	−4.92, 1.98
Maternal Pre-Pregnancy BMI	−1.34	−4.97, 2.29	−2.18	−5.70, 1.33	−1.67	−5.00, 1.66
Education	−1.60	−5.38, 2.18	−2.32	−5.98, 1.33	−1.64	−5.03, 1.75
MVPA	−1.61	−5.45, 2.22	−2.53	−6.19, 1.13	−1.03	−4.63, 2.56
**Fine Motor**						
Primary Model	0.26	−3.55, 4.06	−1.92	−5.66, 1.82	−1.82	−5.43, 1.79

Maternal Age	−0.55	−4.49,3.39	−2.55	−6.34, 1.24	−2.27	−6.12, 1.18
Race	0.27	−3.61, 4.14	−1.95	−5.77, 1.88	−1.85	−5.55, 1.86
Household Income	0.49	−3.61, 4.59	−1.67	−5.63, 2.29	−1.99	−5.73, 1.74
Feeding Type	0.34	−3.56, 4.24	−1.89	−5.72, 1.95	1.83	−5.50, 1.84
Maternal Pre-Pregnancy BMI	0.18	−3.76, 4.13	−1.99	−5.78, 1.80	−1.81	−5.46, 1.83
Education	−0.11	−4.20, 3.97	−2.08	−5.99, 1.82	−2.09	−5.75, 1.57
MVPA	2.00	−1.87, 5.89	−1.27	−5.01, 2.45	−1.19	−4.96, 2.59
**Perception–Action**						
Primary Model	−1.35	−4.08, 1.38	−1.36	−4.09, 1.36	−0.73	−3.36, 1.89
Maternal Age	−1.18	−4.05, 1.69	−1.22	−4.02, 1.59	−0.55	−3.26, 2.16
Race	−1.34	−3.98, 1.30	−1.72	−4.36, 0.91	−1.07	−3.63, 1.48
Household Income	−2.71	−5.53, 0.09	−2.25	−5.02, 0.52	−1.57	−4.21, 1.06
Feeding Type	−1.33	−4.09, 1.44	−1.500	−4.25, 1.25	−0.70	−3.33, 1.93
Maternal Pre-Pregnancy BMI	−1.90	−4.67, 0.87	−1.57	−4.27, 1.14	−0.61	−3.22, 2.00
Education	−1.91	−4.81, 0.99	−1.66	−4.50, 1.18	−0.64	−3.31, 2.04
MVPA	−0.03	−2.80, 2.74	−0.87	−3.57, 1.84	−0.73	−3.54, 2.08
**MVPA**						
**Gross Motor**						
Primary Model	1.90	−1.58, 5.37	0.28	−3.27, 3.84	−1.03	−4.75, 2.68
Maternal Age	1.90	−1.55, 5.35	−0.17	−3.77, 3.43	−0.78	−4.47, 2.92
Race	2.04	−1.46, 5.53	0.89	−2.84, 4.62	−0.52	−4.32, 3.28
Household Income	1.96	−1.57, 5.49	0.19	−3.43, 3.81	−1.16	−4.96, 2.63
Feeding Type	1.81	−1.73, 5.35	0.24	−3.39, 3.87	−0.95	−4.72, 2.82
Maternal Pre-Pregnancy BMI	1.90	−1.49, 5.29	1.15	−2.40, 4.70	−0.15	−3.91, 3.61
Education	2.10	−1.40, 5.59	1.28	−2.36, 4.92	−0.52	−4.26, 3.31
SED	1.39	−2.37, 5.16	−0.15	−3.77, 3.47	−1.23	−5.06, 2.60
**Fine Motor**						
Primary Model	4.33	0.81, 7.84	3.72	0.10, 7.33	2.70	−1.20, 6.60
Maternal Age	4.33	0.85, 7.82	4.36	0.75, 7.96	2.44	−1.45, 6.34
Race	4.34	0.75, 7.92	3.97	0.14, 7.81	3.01	−1.04, 7.05
Household Income	4.08	0.53, 7.63	3.47	0.17, 7.12	1.85	−2.01, 5.70
Feeding Type	4.31	0.72, 7.90	3.73	0.03, 7.42	2.79	−1.22, 6.74
Maternal Pre-Pregnancy BMI	4.33	0.78, 7.87	3.83	0.10, 7.56	2.75	−1.31, 6.82
Education	5.01	1.45, 8.59	4.46	0.72, 8.21	3.31	−0.76, 7.38
SED	5.01	1.20, 8.81	3.53	0.16, 7.22	2.47	−1.55, 6.50
**Perception–Action**						
Primary Model	3.78	1.29, 6.27	2.87	0.27, 5.47	1.42	−1.48, 4.33
Maternal Age	3.78	1.27, 6.28	2.81	0.14, 5.48	1.54	−1.39, 4.46
Race	3.72	1.31, 6.12	2.54	−0.11, 5.19	1.11	−1.76, 3.99
Household Income	3.50	1.04, 5.96	2.75	0.06, 5.30	0.65	−2.25, 3.55
Feeding Type	3.76	1.25, 6.26	2.70	0.07, 5.34	1.60	−1.31, 4.50
Maternal Pre-Pregnancy BMI	3.78	1.31, 6.25	2.56	0.01, 5.24	0.93	−2.05, 3.91
Education	3.86	1.30, 6.42	2.59	−0.15, 5.34	1.05	−1.98, 4.09
SED	3.78	1.07, 6.50	2.73	0.06, 5.41	1.28	−1.71, 4.28

Each potential confounding variable was added to the primary model one at a time (adjusted for age splines only). SED: sedentary behavior; MVPA: moderate-to-vigorous intensity physical activity; BMI: body mass index (kg/m^2). Std. ß: Coefficient representing change in EMQ score per 1 SD change SED or MVPA. SD SED: Trimester 1: 87.2 Trimester 2: 75.8 Trimester 3: 81.2 min. SD MVPA: Trimester 1: 16.6 Trimester 2: 17.3 Trimester 3: 17.3 min.
